# Linking Age Changes in Human Cortical Microcircuits to Impaired Brain Function and EEG Biomarkers

**DOI:** 10.1111/acel.70329

**Published:** 2025-12-14

**Authors:** Alexandre Guet‐McCreight, Shreejoy Tripathy, Etienne Sibille, Etay Hay

**Affiliations:** ^1^ Centre for Addiction and Mental Health Krembil Centre for Neuroinformatics Toronto Ontario Canada; ^2^ Department of Physiology University of Toronto Toronto Ontario Canada; ^3^ Department of Psychiatry University of Toronto Toronto Ontario Canada; ^4^ Institute of Medical Science University of Toronto Toronto Ontario Canada; ^5^ Centre for Addiction and Mental Health Campbell Family Mental Health Research Institute Toronto Ontario Canada; ^6^ Department of Pharmacology and Toxicology University of Toronto Toronto Ontario Canada

## Abstract

Human brain aging involves a variety of cellular and synaptic changes, but how these changes affect brain function and signals remains poorly understood due to experimental limitations in humans, meriting the use of detailed computational models. We identified key human cellular and synaptic changes occurring with age from previous studies, including a loss of inhibitory cells, NMDA receptors, and spines. We integrated these changes into our detailed human cortical microcircuit models and simulated activity in middle age (~50 years) and older (~70 years) microcircuits, and linked the altered mechanisms to reduced spike rates and impaired signal detection. We then simulated EEG potentials arising from the microcircuit activity and found that the emergent power spectral changes due to these aging cellular mechanisms reproduced most of the resting‐state EEG biomarkers seen in human aging, including reduced aperiodic offset, exponent, and periodic peak center frequency. Using machine learning, we demonstrated that the changes to the cellular and synaptic aging mechanisms can be estimated accurately from the simulated EEG aging biomarkers. Our results link cellular and synaptic mechanisms of aging with impaired cortical function and physiological biomarkers in clinically relevant brain signals.

## Graphical abstract

We integrated human data of aging‐related reductions in inhibition, NMDA function, and dendritic spines into detailed models of cortical microcircuits. Simulations showed that the cellular aging mechanisms accounted for many of the age‐related changes seen in human EEG and could be estimated accurately using machine learning from their EEG biomarkers.

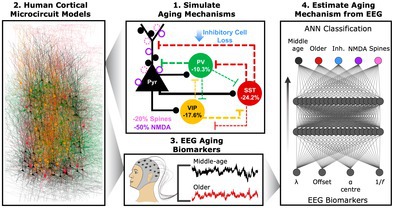

## 1 Introduction

Human aging involves a variety of neurophysiological changes at the cortical microcircuit level, including loss of inhibitory cells (Chen et al. 2023), synaptic composition changes (Pegasiou et al. 2020), and synaptic spine loss (Petanjek et al. 2011). However, the contributions of these changes to age‐related impairments in human brain function, such as executive function and sensory processing (Bucur and Madden 2010), remain poorly understood and result in gaps in diagnosis and treatments. As our ability to study microcircuits in the living human brain is limited, detailed computational models of human brain microcircuits (Yao et al. 2022) offer a powerful tool to mechanistically link microcircuit changes to altered brain function and clinically relevant brain signals such as electroencephalography (EEG) in aging (Donoghue et al. 2020).

A variety of age‐related mechanisms have been identified across scales and species, and several key cellular and microcircuit changes have been characterized in detail in the human brain. Transcriptomic studies reported an association between age and differences in cell type proportions (Chen et al. 2023), including reductions in somatostatin‐(SST) and vasoactive intestinal polypeptide (VIP) expressing interneurons. Human layer 2/3 pyramidal neurons in older individuals also exhibited reduced N‐methyl‐D‐aspartate (NMDA) GluN2A/B protein levels and NMDA‐associated synaptic currents (Pegasiou et al. 2020). Starting from early adulthood, there was also a steady decline of dendritic spine counts in human pyramidal neurons (Petanjek et al. 2011; Boros et al. 2019), in particular thin spines and immature spines (filopodia) (Barzó et al. 2024). Previous studies also suggest inter‐dependencies between these mechanisms, whereby filopodia were shown to mainly contain NMDA receptors and lack AMPA receptors (Barzó et al. 2024; Vardalaki et al. 2022), boosting SST‐mediated inhibition recovered pyramidal neuron spine counts in aged rodents (Prevot et al. 2021), and long‐term dampening of SST‐mediated inhibition reduced NMDA‐mediated excitation (Nuwer et al. 2023).

These mechanisms have been linked to human and rodent age‐related neurological disease and cognitive decline (Prevot et al. 2021; Gabitto et al. 2024). Using novel pharmacology to restore loss of SST cell signaling in aged rodents, which can occur due to increased neuroinflammation (Rezaei et al. 2024; Gavilán et al. 2007), hypermethylation (McKinney et al. 2015) and endoplasmic reticulum stress (Tomoda et al. 2022), was sufficient to recover working memory (Prevot et al. 2021). NMDA loss has been linked to age‐dependent decline of slow inward currents, which modulates spike timing‐dependent plasticity with age (Csemer et al. 2023). In addition, pyramidal neuron spine loss was greater in cognitively impaired older rats compared to unimpaired older and younger rats (Allard et al. 2012).

Given the limitations in collecting in vivo neuronal and microcircuit data from living humans, non‐invasive EEG recordings remain one of the most useful methods for measuring human neuronal activity in health and disease. Several previous studies found consistent and robust age‐associated changes in human EEG power spectral features, whereby power spectral decomposition into aperiodic (broadband) and periodic components showed reductions in aperiodic slope and power (Donoghue et al. 2020; Merkin et al. 2023) and reductions in periodic peak alpha frequency and power (Donoghue et al. 2020; Merkin et al. 2023; Park et al. 2024; Smith et al. 2023). Despite the robust findings about EEG biomarkers in aging, the links to the underlying changes in neuronal and microcircuit mechanisms remain unknown.

Age‐associated cognitive decline in older adults includes slower cortical processing (Bucur and Madden 2010), reduced working memory capacity (Peich et al. 2013), and reduced discrimination acuity (Legge et al. 2008, 2019). Studies in monkeys have also shown that a decline in working memory with age was associated with reduced baseline and response spike rates in the prefrontal cortex (Wang et al. 2011). Correspondingly, age‐associated declines in spike rates were reported in multi‐electrode array data obtained from human cortical slices (Guet‐McCreight et al. 2022). However, the link to underlying mechanisms remains unclear.

To overcome experimental limitations in linking altered microcircuit and cellular mechanisms to cognitive impairment and EEG in human aging, we integrated aging mechanisms from human cellular studies into our previous detailed models of human cortical microcircuits. Using these models, we simulated EEG and microcircuit spiking activity to delineate the contributions of cellular aging to microcircuit function, signal integration, and EEG biomarkers of aging.

## 2 Results

**FIGURE 1:**
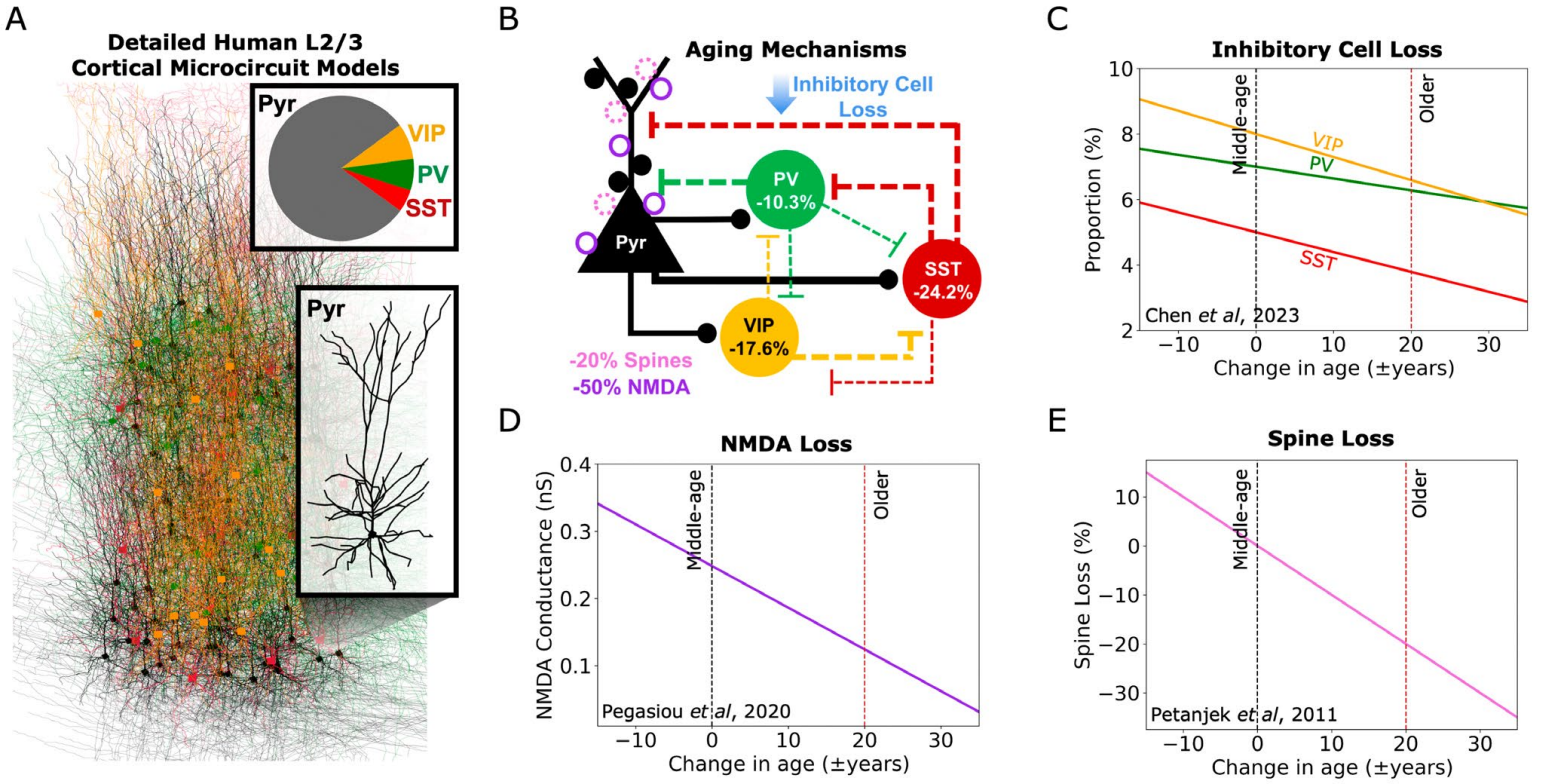
Modeling multiple microcircuit cellular and synaptic mechanisms of human aging. (A) Illustration of the detailed human L2/3 microcircuit model, which included four key neuron types with human cell proportions, detailed morphologies, firing, and synaptic properties. (B) Schematic of the aging microcircuit model, showing connectivity between neuron types and integration of human aging mechanisms data including loss of inhibitory cells, NMDA GluN2A/B protein levels, and spines due to +20 years aging. (C–E) Data‐driven relationship between age and inhibitory cell proportion loss (C), NMDA conductance loss (D), and percent spine loss (E). Dashed lines show middle‐age and older (aged +20 years) values.

We simulated the effect of aging mechanisms on detailed models of prototypical human cortical microcircuits, representing microcircuits of middle‐aged (~50 years old) individuals (Figure 1A). The microcircuits included four key neuron types (Pyr neurons and SST, PV, and VIP interneurons) constrained with human data of their firing properties, morphologies, cell proportions, synaptic properties, and connection probabilities (Figure 1A,B). We integrated cellular and synaptic aging mechanisms from human studies to simulate microcircuits of older individuals. These included loss of inhibitory cells derived from transcriptomic data (Chen et al. 2023), loss of Pyr neuron NMDA receptors derived from protein level and synaptic current data (Pegasiou et al. 2020), and loss of Pyr neuron spines derived from imaging data (Petanjek et al. 2011), estimated over a 20‐year span (i.e., ~50 vs. ~70 years; Figure 1B–E). Because the relationship between mechanism changes and age was approximately linear, we modeled the changes linearly as proportional changes in the model's SST, VIP, and PV inhibitory cell counts (Figure 1C), the NMDA conductance onto postsynaptic Pyr neurons (Figure 1D), and the connection probability and passive parameters of Pyr neurons (reflecting spine loss; Figure 1E).

**FIGURE 2:**
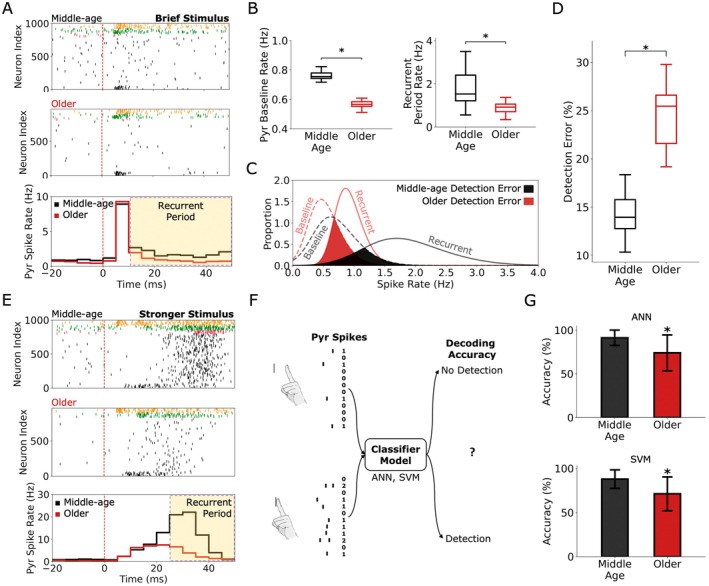
Human aging microcircuit mechanisms reduce spike rates and impair signal processing. (A) Example raster plots of middle‐age and older microcircuits at baseline and in response to a brief stimulus (top), and average peri‐stimulus time histograms (PSTH, bottom). Shaded yellow area denotes the time range for the recurrent activity period. Dashed line shows stimulus time. (B) Mean Pyr neuron spike rates at baseline (left) and recurrent response (right) for middle‐age and older microcircuits (20 randomized each). (C) Distribution of firing rates in the baseline and recurrent response activity periods for the middle‐age and older microcircuits. Signal detection error was measured by the percent overlap between the baseline and recurrent period response rate distributions (shaded area). (D) Signal detection error rate for middle‐age and older microcircuits (bootstrapped 1000 permutations). (E) Example middle‐age and older microcircuit raster plots (top) and mean PSTH (bottom) in response to a stronger and de‐correlated stimulus. (F) Schematic of how decoding accuracy was assessed. Spike trains in 50 ms baseline and recurrent period response windows (40 windows total per condition) were summed into spike count vectors (left) and inputted into a classifier model. (G) Decoding accuracy when using an ANN (top) and a SVM (bottom) for the decoding (*n* = 100 randomized classifiers). Asterisks denote significant *t*‐tests and Cohen's *d* > 0.5 of older microcircuits compared to middle‐age microcircuits. For box‐and‐whisker plots, boxes show interquartile range (IQR), middle lines show the medians, and whiskers show 1.5× the IQR.

We simulated the middle‐aged (~50 years) and older (~70 years) microcircuits, with randomized circuitries to mimic between‐person variability, at baseline activity and in response to a brief stimulus (Figure 2A). Older microcircuits had reduced spike rates during baseline (0.76 ± 0.04 Hz vs. 0.57 ± 0.03 Hz, *p* = 1.08e‐14, Cohen's *d* = −6.0) and post‐stimulus recurrent activity (2.53 ± 0.76 Hz vs. 1.74 ± 0.27 Hz, *p* = 8.20e‐4, Cohen's *d* = −1.3; Figure 2B). The dampened activity resulted in higher signal detection errors in older microcircuits, based on the readout of recurrent activity vs. baseline as measured by the overlap between the spike rate distributions (14.3% ± 2.2% vs. 24.9% ± 3.8%, *p* = 1.36e‐8, Cohen's *d* = 3.3; Figure 2C,D). We further simulated activity in response to a stronger stimulus with enhanced recurrent activity and found a larger effect of the aging mechanisms in reducing post‐stimulus recurrent activity (7.01 ± 3.49 Hz vs. 2.12 ± 0.53 Hz, *p* = 5.64e‐6, Cohen's *d* = −1.9; Figure 2E). With this stimulus, an increase in signal detection errors was similarly seen using classifier models (ANN or SVM) to distinguish between baseline and recurrent response activity (Figure 2F). We found that classification accuracy decreased with older microcircuits (ANN: 74.0% ± 20.5% vs. 91.2% ± 8.8%, *p* = 1.20e‐11, Cohen's *d* = −1.1; SVM: 71.2% ± 19.1% vs. 88.0% ± 10.5%, *p* = 7.40e‐11, Cohen's *d* = −1.1; Figure 2G).

**FIGURE 3:**
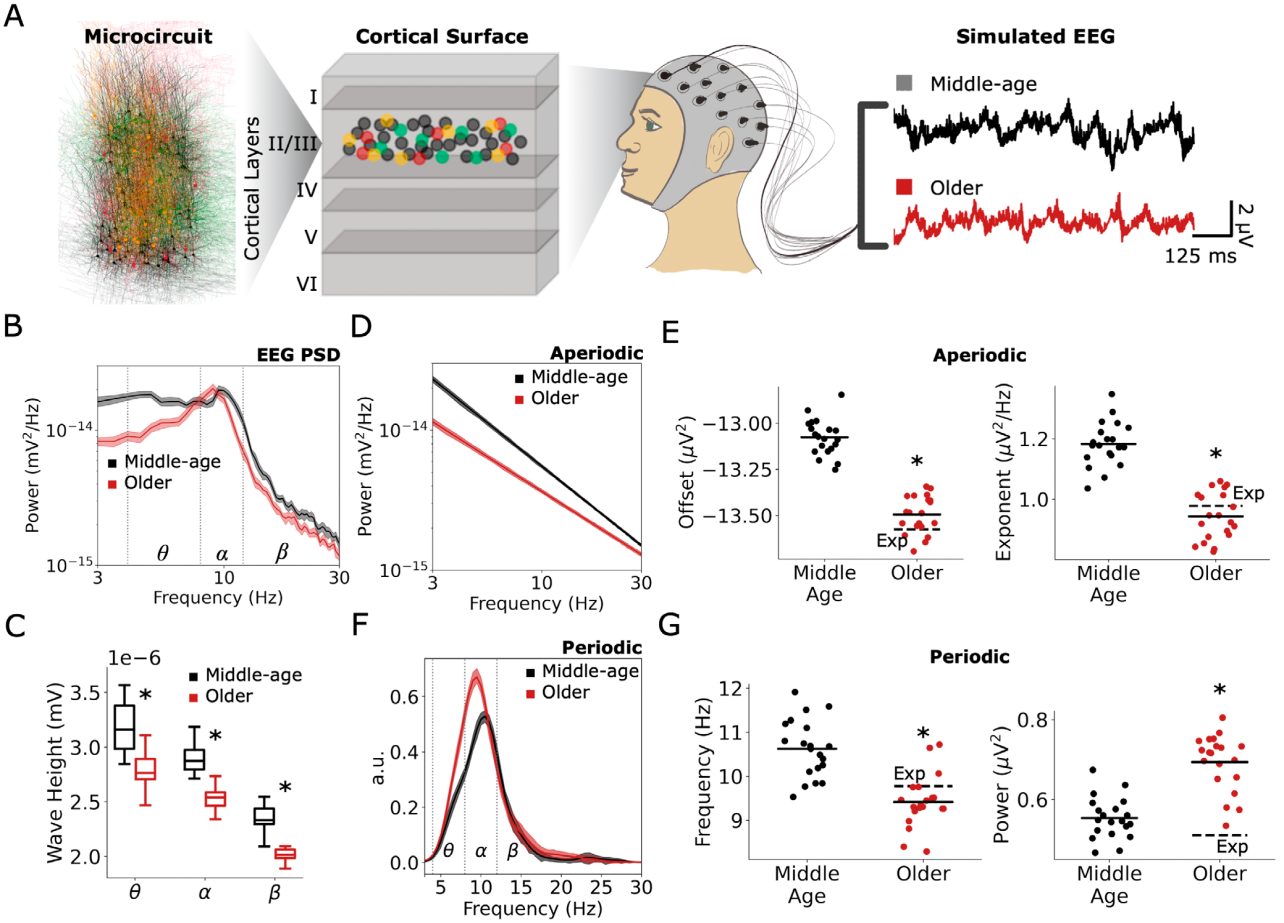
Human aging microcircuit mechanisms account for key EEG biomarkers. (A) Schematic of simulated EEG signals generated from the human microcircuit models. (B) Power spectral density (PSD) of simulated EEG from middle‐age and older microcircuit models (bootstrapped mean and 95% confidence intervals). Inset—PSD in log scale. (C) Wave height extracted from oscillation events and separated by frequency bands. (D, E) Aperiodic components of the PSD (D) and associated parameters (E, left: offset, right: exponent; dots correspond to *n* = 20 simulated microcircuits in each age condition). (F–G) Periodic components of the PSD (F) and associated parameters (G, left: peak centre frequency, right: peak amplitude). In E and G, the dashed lines (Exp) indicate the relative expected changes based on previously reported changes in human aging (Merkin et al. 2023). Asterisks and box‐and‐whisker plots are as described in Figure 2. *n* = 20 randomized microcircuit simulations per condition.

We next assessed the effect of the aging mechanisms on the simulated EEG from the microcircuit models (Figure 3A), in terms of EEG power spectral density (PSD, Figure 3B), and found a left shift of peak α frequency (see periodic component analysis below) and reduced power in *θ*, *α*, β frequency bands as measured by AUC (*θ*: 0.066 ± 0.007 μV^4^/Hz^2^ vs. 0.034 ± 0.004 μV^4^/Hz^2^, *p* = 2.17e‐12, Cohen's *d* = −5.7; *α*: 0.065 ± 0.005 μV^4^/Hz^2^ vs. 0.057 ± 0.007 μV^4^/Hz^2^, *p* = 2.50e‐4, Cohen's *d* = −1.3; *β*: 0.081 ± 0.006 μV^4^/Hz^2^ vs. 0.063 ± 0.006 μV^4^/Hz^2^, *p* = 4.11e‐10, Cohen's *d* = −3.0). Correspondingly, in performing a wavelet‐based spectrogram event analysis we found decreased wave heights (*θ*: 3.18 ± 0.22 nV vs. 2.79 ± 0.16 nV, *p* = 2.56e‐7, Cohen's *d* = −2.0; *α*: 2.89 ± 0.12 nV vs. 2.53 ± 0.12 nV, *p* = 2.05e‐8, Cohen's *d* = −3.0; *β*: 2.34 ± 0.12 nV vs. 2.03 ± 0.09 nV, *p* = 1.29e‐8, Cohen's *d* = −3.0, Figure 3C).

To better analyze the changes in PSD, we decomposed it into aperiodic and periodic components. The aperiodic component (Figure 3D) in older microcircuits exhibited decreased offset (−13.08 ± 0.10 μV^2^ vs. −13.49 ± 0.10 μV^2^, *p* = 1.63e‐11, Cohen's *d* = −4.2, Figure 3E) and exponent (1.18 ± 0.07 μV^2^/Hz vs. 0.94 ± 0.08 μV^2^/Hz, *p* = 3.42e‐9, Cohen's *d* = −3.1; Figure 3E), consistent with changes seen experimentally (Merkin et al. 2023). The periodic component of the PSD (Figure 3F) exhibited decreased peak centre frequency (10.63 ± 0.64 Hz vs. 9.42 ± 0.59 Hz, *p* = 4.68e‐7, Cohen's *d* = −1.9, Figure 3G), consistent with changes seen experimentally (Merkin et al. 2023). However, it also exhibited increased periodic peak amplitudes (0.55 ± 0.05 vs. 0.69 ± 0.07, *p* = 1.80e‐8, Cohen's *d* = 2.24; Figure 3G), which were inconsistent with the expected decrease previously reported. Importantly, these changes in EEG emerged simply from implementing the cellular and synaptic aging mechanisms and were not explicitly optimized or tuned for.

**FIGURE 4:**
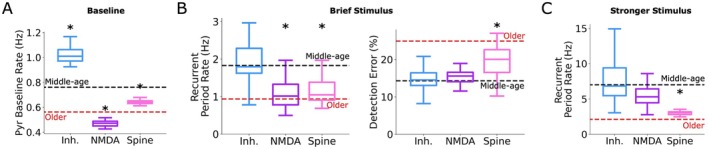
Individual aging cellular and synaptic mechanisms have distinct effects on microcircuit baseline and response activity. (A) Mean baseline Pyr neuron spike rates for older microcircuits with each aging mechanism implemented separately, compared to middle‐age (black dashed lines) and combined mechanisms (red dashed lines). (B) Recurrent response to a brief stimulus (left) for each aging mechanism and corresponding signal detection errors (right). (C) Recurrent response to a stronger stimulus for each aging mechanism. *n* = 20 randomized microcircuit simulations per condition. Box‐and‐whisker plots are as described in Figure 2.

We characterized the effects of individual aging cellular and synaptic mechanisms on microcircuit spiking. While both NMDA loss and spine loss reduced baseline spike rates relative to middle‐aged microcircuits (0.47 ± 0.02 Hz, *p* = 2.31e‐19, Cohen's *d* = −10.0; 0.64 ± 0.03 Hz, *p* = 3.70e‐10, Cohen's *d* = −3.5, Figure 4A), inhibitory cell loss increased baseline spike rates (1.02 ± 0.05 Hz, *p* = 1.38e‐15, Cohen's *d* = 5.7). In response to a brief stimulus, both NMDA loss and spine loss decreased recurrent response period rates (1.85 ± 0.41 Hz, *p* = 1.91e‐3, Cohen's *d* = −1.1; 1.97 ± 0.27 Hz, *p* = 0.005, Cohen's *d* = −0.9, Figure 4B, left), with no significant effect of inhibitory cell loss, but only spine loss worsened signal detection error (19.76% ± 3.95%, *p* = 1.58e‐4, Cohen's *d* = −1.6; Figure 4B, right). In response to the stronger stimulus, only spine loss decreased recurrent response rate relative to middle‐aged microcircuits (3.00 ± 0.48 Hz, *p* = 1.01e‐4, Cohen's *d* = −1.6; Figure 4C), and none of the mechanisms had an effect of decreasing classification accuracy individually.

**FIGURE 5:**
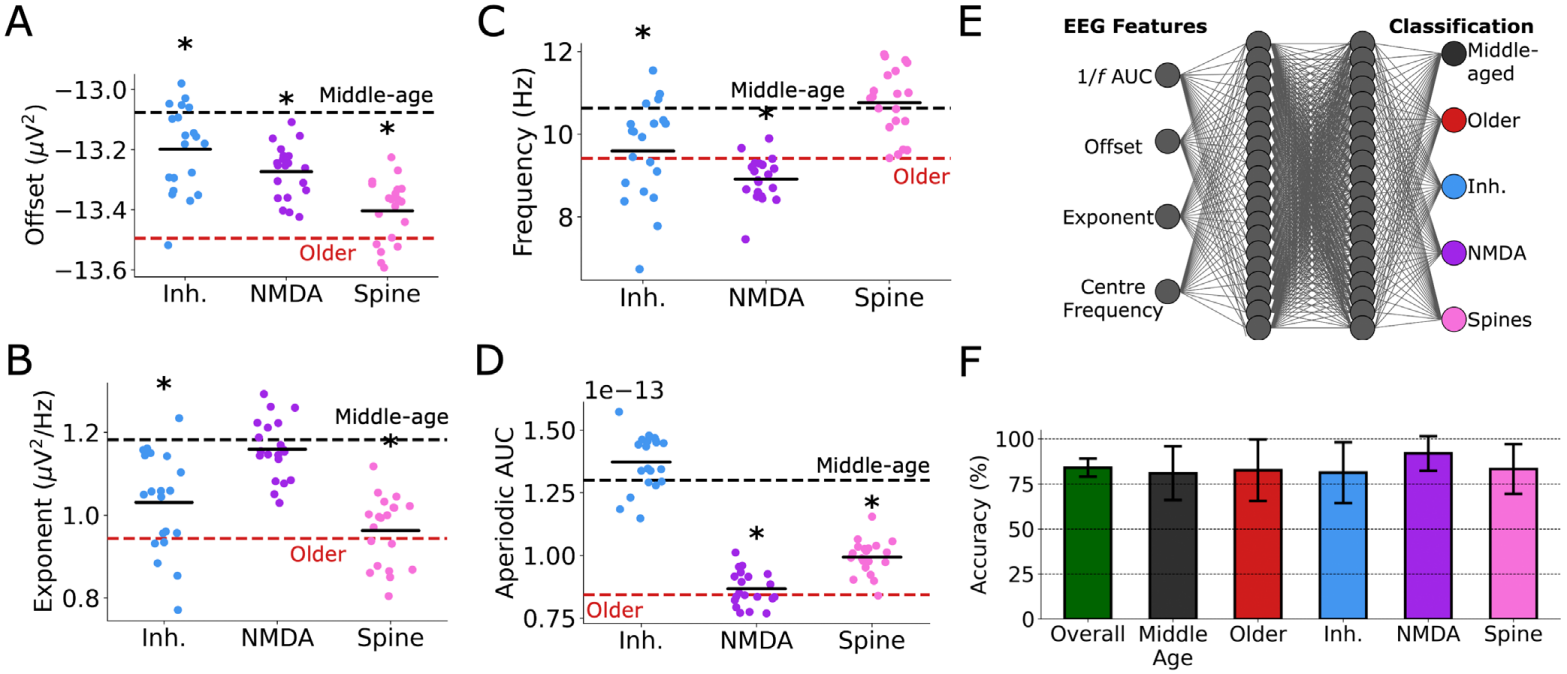
EEG biomarkers of microcircuit aging cellular and synaptic mechanisms. (A–D) Aperiodic offset (A), exponent (B), periodic peak centre frequency (C), and 1/*f* AUC parameters of each aging mechanism compared to middle‐age (black dashed lines) and combined mechanisms (red dashed lines). All asterisks denote significant paired *t*‐tests (*p* < 0.05) with effect sizes > 0.5, when compared to middle‐age microcircuits (black asterisks). *n* = 20 randomized microcircuit simulations per condition. Box‐and‐whisker plots are as described in Figure 2. (E) ANN architecture for predicting changes in the cellular and synaptic aging mechanisms from key EEG features. (F) Test classification accuracies overall and per condition. Accuracy was computed across *n* = 50 ANN using permutations of 60/10/30% train/validate/test datasets.

We next characterized the contributions of individual aging mechanisms to the above EEG biomarkers. All three mechanisms decreased aperiodic offset relative to middle‐aged microcircuits (inhibitory cells: −13.08 ± 0.10 μV^2^, *p* = 9.42e‐3, Cohen's *d* = −1.0; NMDA: −13.27 ± 0.09 μV^2^, *p* = 2.59e‐6, Cohen's *d* = −2.1; spine: −13.40 ± 0.10 μV^2^, *p* = 2.51e‐9, Cohen's *d* = −3.2, Figure 5A). However, only inhibitory cell loss and spine loss decreased aperiodic exponents (inhibitory cells: 1.03 ± 0.12 μV^2^/Hz, *p* = 3.01e‐4, Cohen's *d* = −1.5; spine: 0.96 ± 0.08 μV^2^/Hz, *p* = 1.63e‐8, Cohen's *d* = −2.8, Figure 5B). In contrast, only inhibitory cell loss and NMDA loss decreased periodic peak centre frequency (inhibitory cells: 9.60 ± 1.16 Hz, *p* = 1.70e‐3, Cohen's *d* = −1.1; NMDA: 8.92 ± 0.52 Hz, *p* = 5.96e‐11, Cohen's *d* = −2.8, Figure 5C). We additionally characterized the effect on aperiodic area‐under‐the‐curve (1/*f* AUC), for which only NMDA loss and spine loss decreased relative to middle‐aged microcircuits (middle‐aged: 0.130 ± 0.011 μV^4^/Hz^2^; NMDA: 0.087 ± 0.007 μV^4^/Hz^2^, *p* = 1.17e‐12, Cohen's *d* = −4.6; spine: 0.099 ± 0.007 μV^4^/Hz^2^, *p* = 9.49e‐9, Cohen's *d* = −3.2, Figure 5D). 1/*f* AUC showed a similar direction and magnitude of the aging mechanisms' effects as the baseline spike rates (Figures 4A and 5D). In line with this, across conditions we found that 1/*f* AUC correlated strongly with baseline spike rates (Pearson *R* = 0.85, *p* = 9.59e‐30).

Given the differential effects of age‐related cellular and synaptic mechanisms on EEG, we assessed whether an artificial neural network (ANN) could classify changes in the cellular and synaptic aging mechanisms based on our simulated EEG biomarkers (Figure 5E). We found high classification accuracy for individual or combined mechanisms in older microcircuits (mean accuracy: 84.1% ± 5.0%, range: 73.3%–93.3%; Figure 5F). The ANN performed this classification by relying mainly on exponent (SHAP: 0.09 ± 0.02), 1/*f* AUC (SHAP: 0.09 ± 0.02), and offset (SHAP: 0.08 ± 0.03), with a smaller contribution from centre frequency (SHAP: 0.05 ± 0.02).

## 3 Discussion

In this work, we integrated aging mechanisms into detailed models of human cortical microcircuits and showed that key cellular and circuit human aging mechanisms can account for age‐associated changes in spiking and EEG biomarkers. Importantly, the EEG effects emerged from simply implementing the cellular and synaptic aging mechanisms and were not explicitly tuned for. We further demonstrated that the cellular and synaptic aging changes could be estimated from simulated EEG power spectral biomarkers using artificial neural networks. Our findings overcome challenges in linking cellular and synaptic aging mechanisms with impaired cortical function and aging biomarkers in brain signals, which can enable a more mechanistic stratification of older individuals with age‐associated cognitive decline.

The simulated aging EEG effects that we found reproduce most of the biomarkers that have been observed and replicated in several studies, including reduced aperiodic exponent, offset, and periodic peak center frequency (Donoghue et al. 2020; Merkin et al. 2023). Our results are also in line with previous work that showed accurate estimation of age from EEG (Engemann et al. 2022; Khayretdinova et al. 2022). Here we provide the first link between the EEG effects and human cellular and synaptic aging mechanisms and show that each mechanism generated unique effects. In line with reduced aperiodic parameters due to inhibitory cell loss, boosting inhibition with either pentobarbital or diazepam in rodents has been shown to increase aperiodic exponent and offset (Salvatore et al. 2024). This was also seen in macaques during propofol administration, and was predicted by computational simulations of reduced E/I balance via increased inhibition (Gao et al. 2017). Changes in aperiodic exponents with age have also been linked to cognitive decline (Smith et al. 2023), a relationship that is influenced by level of education (Montemurro et al. 2024). In line with our effects of inhibitory cell and NMDA loss on alpha peak center frequency, blocking inhibition with picrotoxin or blocking NMDA with ketamine both lead to reduced peak periodic frequencies in the 6–10 Hz range in rodents (Salvatore et al. 2024). Decreased alpha peak frequencies in aging have also been associated with working memory deficits (Cesnaite et al. 2023). While no studies to date have directly characterized the effects of dendritic spine changes on EEG, spine density is decreased in rodent medial prefrontal cortex during corticosterone administration (Anderson et al. 2016), which has also been associated with decreased aperiodic exponent and offset (Bergosh et al. 2024), in line with the effects of spine loss in our models. Interestingly, the alignment in the direction of effects across mechanisms between 1/*f* AUC and baseline spike rate that we found suggests that the dynamics of both measures is similarly sensitive to changes in these underlying neurophysiological mechanisms. In addition, the strong correlation between 1/*f* AUC and baseline spike rate indicates that the simulated resting state activity involves mostly aperiodic, noisy spiking.

The aging mechanisms led to reduced baseline and response spike rates, in line with effects reported in human cortical slices (Guet‐McCreight et al. 2022) and in vivo in monkeys during a working memory task (Wang et al. 2011). These spiking changes resulted in worsened signal detection errors, in agreement with impaired cognitive performance during sensory discrimination in older subjects (Legge et al. 2019). Baseline spike rates were increased by inhibitory neuron loss, consistent with our previous work on the effects of SST inhibition loss (Yao et al. 2022), and decreased by NMDA and spine loss, as expected from reduced excitation onto Pyr neurons (Eyal et al. 2018; Hay and Segev 2015). While inhibitory neuron loss had no effect on response rates (consistent with our previous work (Yao et al. 2022)), both NMDA and spine loss reduced recurrent responses to brief stimuli, consistent with previous findings on excitatory effects during brief thalamocortical stimulation (Hay and Segev 2015) and ketamine application in vivo (Kauvar et al. 2025). Conversely, spine loss but not NMDA loss reduced recurrent responses to stronger stimuli, possibly due to dendritic saturation effects (i.e., reduced driving force) with larger stimuli (Eyal et al. 2018; Guet‐McCreight and Skinner 2019) causing smaller differences in NMDA output at higher activation levels. We did not disentangle the effects of SST, PV, and VIP interneuron loss, but the EEG changes we report due to inhibitory interneuron loss match more distinctly with EEG changes seen during SST inhibition loss rather than PV inhibition loss (Mazza et al. 2023), which is in line with the greater magnitude of SST loss that we simulate in our aging models.

While the changes in aging mechanisms that we modeled accounted for several key features of aging EEG, none of the mechanisms alone or together replicated the decrease in periodic peak power seen previously (Donoghue et al. 2020; Merkin et al. 2023), suggesting the involvement of other aging mechanisms. Changes in ion channel balances such as those we reported previously in human L5 Pyr neurons (Guet‐McCreight et al. 2022) may contribute to a decrease in periodic peak power. Age‐related EEG changes may also be affected by non‐neuronal mechanisms, such as increased glial cell activity (Chen et al. 2023), changes in cardiac activity (Schmidt et al. 2025), genetic risk factors for dementia (Smith et al. 2023), or other cardiovascular factors (Smith et al. 2023). In addition, while the experimental aging mechanism reductions were approximately linearly related with age in young adults to older individuals, future experimental studies with larger sample sizes may further characterize the relationships and any potential non‐linearities, which could refine our models of cellular and microcircuit aging. The changes in periodic peak power with age may also involve components not currently included in the model, such as layer 5, which contributes to beta rhythm generation (Guet‐McCreight et al. 2022; Roopun et al. 2006) and may shift dynamics away from layer 2/3 alpha dominance, thus influencing aging effects on spectral power. Periodic peak power may also be influenced by active dendritic properties (Yao et al. 2025; Reimann et al. 2013), whose presence can impact the effects of changes in synaptic input (Hay and Segev 2015; Guet‐McCreight and Skinner 2020) and thus affect microcircuit spectral dynamics. Given the low incidence of beta events in our layer 2/3 models, these were not well suited to capture changes in beta frequency oscillations, which increase in peak power and decrease in peak centre frequency with age (Park et al. 2025). While we simulated a generic prototypical human cortical microcircuit and studied general stimulus response (which can apply to either sensory or higher order regions), future studies may benefit from modeling a specific area of cortex, such as sensory or prefrontal cortex (Rosanally et al. 2024) to study particular age‐related impairments. In addition, exploring the effects of cellular and synaptic aging over more time points could provide additional insights into how these mechanisms impact EEG and microcircuit function over time.

While we tested the accuracy of ANN trained for mechanistic classification, another approach would be to train ANN for estimating the level of cellular aging, which would require more biophysical simulations of different severity levels for each mechanism. Although the ANN we used had good classification accuracy using two hidden layers, future studies could conduct a more comprehensive search through network architecture and feature sets to further improve the accuracy or establish the utility of features. It will be of particular interest for future studies to apply our in silico‐trained artificial neural networks to estimate cellular aging for human subjects from their EEG data and assess the correspondence with cognitive impairments. While confounds such as head model differences, medication effects, and cardiovascular influences need to be taken into account (Chinoy et al. 2014; Barbaux et al. 2025; Kumral et al. 2020; Mullinger et al. 2013), this method will enable a more mechanistic stratification, improved diagnosis, and establish mechanism‐based target treatments in aging.

## 4 Methods

### 4.1 Models of Human Cortical Microcircuits

We used our previous morphologically‐ and biophysically‐detailed models of human L2/3 cortical microcircuits (Yao et al. 2022). The models were comprised of 1000 neurons belonging to key neuron types (80% Pyr, 5% SST, 7% PV, and 8% VIP as estimated from RNA‐seq data (Hodge et al. 2019)) distributed across a 500 × 500 × 950 μm^3^ volume. Neuron firing and synaptic connection properties were fitted to human data, including Pyr → Pyr (Seeman et al. 2018), SST → Pyr apical dendrites (Obermayer et al. 2018) and PV → Pyr basal dendrites (Komlósi et al. 2012). Synapses were modeled using presynaptic short‐term plasticity parameters for vesicle‐usage, facilitation, and depression, and separate rise and decay parameters for the AMPA and NMDA components of excitatory synapses (τ_rise,NMDA_ = 2 ms; τ_decay,NMDA_ = 65 ms; τ_rise,AMPA_ = 0.3 ms; τ_decay,AMPA_ = 3 ms; τ_rise,GABA_ = 1 ms; τ_decay,GABA_ = 10 ms) (Hay and Segev 2015; Mäki‐Marttunen et al. 2019; Fuhrmann et al. 2002). For a complete list of data provenance in our models, please refer to our previous work (Yao et al. 2022). Simulations were run using NEURON 7.7 (Carnevale and Hines 2006) and LFPy 2.0.2 (Python 3.7.6) (Hagen et al. 2018) on SciNet parallel computing (Ponce et al. 2019). Our microcircuit model represented a prototypical cortical microcircuit of a middle‐aged individual (~50 years), given the diverse human data (morphology, proportions, electrophysiology, and synaptic connectivity) with which the model was constrained involved ages spanning from 18 to 75 years overall (Hodge et al. 2019; Seeman et al. 2018; Hawrylycz et al. 2012; Chameh et al. 2021), and cell proportions from human data with a median age of 45 years (Hodge et al. 2019). We compute all age‐related mechanistic changes in terms of relative years, including reduced inhibitory cells (Chen et al. 2023), reduced NMDA conductance (Pegasiou et al. 2020), and reduced spines (Petanjek et al. 2011). In all cases, we based our model aging mechanisms on human data changes over +20 years (i.e., ~70‐year microcircuits). For each condition, we simulated 20 randomized microcircuits.

### 4.2 Simulating Age‐Dependent Inhibitory Interneuron Loss

For reduced inhibitory cells with age, we analyzed cell type proportions starting from human single‐cell RNA‐seq data (MSSM and McLean cohorts, age range: 24–74 years, *n* = 71, 30 females) (Chen et al. 2023) to extract age regression coefficients and intercepts for each interneuron type in our model (SST, VIP, PV). From these, we derived a loss of 1.21% SST interneurons, 0.88% VIP interneurons, and 0.52% PV interneurons per year, and thus 24.2%, 17.6%, and 10.3% loss of each interneuron type respectively over 20 years aging from middle age to older microcircuits.

### 4.3 Simulating Age‐Dependent NMDA Loss

For reduced NMDA conductance with age, we estimated a 2.5% decline per year in human L2/3 Pyr neuron GluN2A/B protein levels from western blot data (age range: 21–71 years, *n* = 17, 9 females) (Pegasiou et al. 2020), and thus a 50% decline for aging by 20 years. We translated this as a 50% loss of NMDA conductance in our microcircuit models.

### 4.4 Simulating Age‐Dependent Spine Loss

We estimated a 1% spine loss per year in human Pyr neurons from rapid Golgi staining data (age ranges included: 20–91 years; *n* = 18, 4 females) (Petanjek et al. 2011), starting from adulthood (~20 years). We translated this as a 20% loss of Pyr → Pyr connection probability over 20 years aging between the middle‐aged and older microcircuits.

Additionally, we modeled the resulting loss of dendritic surface area by reduced dendritic passive conductance (*G*_pas_) and membrane capacitance (*c*_*m*_). In humans, spine loss with age is mostly due to a loss of thin spines, which, in older individuals, comprise approximately 25% of spines (Barzó et al. 2024). Given a 1% loss over 20 years, we thus estimated that thin spines in middle‐aged individuals comprise approximately 31.25% of spines. Given a total number of 8169 spines in young monkey Pyr neurons (Coskren et al. 2015), and assuming a thin spine length of 1.2 μm (Dumitriu et al. 2010), a tip diameter of 0.35 μm (Dumitriu et al. 2010), and a truncated cone shape with a half base‐to‐tip diameter ratio, we computed the total surface area due to thin spines to be approximately 2782.57 μm^2^. Given a total spine surface area of 11,954 μm^2^ and a total dendritic surface area of 20,410 μm^2^ (Coskren et al. 2015), we computed the proportion of surface area due to thin spines in middle age to be 8.6% of the total dendritic and spine surface area. This yielded a reduction of 1.72% in dendritic (basal + apical) *G*_pas_ and *c*_*m*_ over 20 years of aging between middle age and older microcircuits.

### 4.5 Microcircuit Baseline and Response Spiking Activity

We simulated baseline microcircuit activity as described previously (Guet‐McCreight et al. 2024), where we provided background input to the microcircuit with excitatory Ornstein‐Uhlenbeck (OU) point processes (Destexhe et al. 2001). Independent excitatory OU point processes were placed midway along the length of each dendritic arbor, and for Pyr neuron models, we placed 5 additional OU processes at 10%, 30%, 50%, 70%, 90% of the apical length. We scaled up the mean and standard deviation of each OU conductance exponentially with relative distance from soma (ranging from 0 to 1) to normalize their effect.

For the brief stimulus, we used previous models of response rates (Yao et al. 2022), using excitatory AMPA/NMDA synapses with the same synaptic dynamics as the cortical excitatory synapses. We stimulated the basal dendrites of 55 Pyr neurons, with a 2–4 ms delay post‐stimulus and a conductance of 4 nS. We also stimulated 35 PV interneurons with a delay of 2–2.5 ms and a conductance of 2 nS. VIP interneurons were stimulated in two groups and phases: early (65 VIP interneurons, delay = 2–2.5 ms, conductance = 2.8 nS) and late (80 VIP interneurons, delay = 7–12 ms, conductance = 2.2 nS).

For the stronger stimulus, we modified the brief stimulus in several ways to promote recurrent activity patterns, by reducing feedforward inhibition (Kim et al. 2016), increasing disinhibition (Lee et al. 2013; Pi et al. 2013) and prolonging excitatory stimulation (Kamiński et al. 2017; Zhang et al. 2025). We first removed stimulation to PV interneurons to reduce feedforward inhibition. We also increased disinhibition by increasing the stimulus *G*_max_ for early VIP interneurons from 2.8 to 5.6 nS and late VIP interneurons from 2.2 to 4.4 nS. Lastly, we prolonged excitatory stimulation by increasing the Pyr neuron stimulus delay range from 2–4 to 2–22 ms, doubled the number of excitatory synapses to Pyr neurons, and compensated by reducing the *G*_max_ for Pyr neurons from 4 to 2 nS.

### 4.6 Signal Detection Metrics and Decoding Accuracy

Similarly to previous work where we computed probabilities of failed and false detections (Yao et al. 2022), we calculated signal detection errors as the percent overlap in the distributions of Pyr neuron firing rates at baseline (computed using a 40 ms sliding window, sliding in 1 ms intervals, over a 3 s pre‐stimulus period) and the distribution of firing rates during the recurrent activation period in response to the brief stimulus (calculated across 20 stimulus presentations, in the 10–50 ms period post‐stimulus).

To assess the decoding accuracy of recurrently activated Pyr neurons in response to a stronger stimulus, we summed the spikes of each Pyr neuron during the baseline period (50 ms pre‐stimulus) and the recurrent activation period (25–75 ms period post‐stimulus). The summed spike trains during the baseline or response periods of each Pyr neuron were then used as input features into classifier models available in the scikit‐learn Python module. The classifier models were trained on 70% of the pre‐/post‐stimulus windows to distinguish baseline (noise) from recurrent response (signal), and were then tested on the remaining 30% of the data. To compute accuracy statistics, we used 100 permutations of train/test subsets.

### 4.7 Simulated Microcircuit EEG and Power Spectral Analysis

As in previous work (Mazza et al. 2023), we simulated resting‐state EEG from our microcircuit models in LFPy 2.0.2 (Python 3.7.6) using a four‐sphere volume conductor model (representing gray matter, cerebrospinal fluid, skull, and scalp with radii of 79, 80, 85, and 90 mm, respectively). The conductivity for each sphere was 0.047, 1.71, 0.02, and 0.41 S m^−1^, respectively (Mazza et al. 2023; McCann et al. 2019). We computed power spectral density (PSD) using Welch's method (Welch 1967) from the Scipy python module with 2s time windows. We also decomposed the EEG power spectra (in the 3–30 Hz range) into periodic and aperiodic components using the FOOOF toolbox (Donoghue et al. 2020). The aperiodic component was a 1/f function parameterized by vertical offset and exponent parameters. We fitted the periodic oscillatory component with up to 3 Gaussian peaks defined by center frequency, bandwidth (min: 2 Hz, max: 6 Hz), and power magnitude (relative peak threshold: 2, minimum peak height: 0) (Mazza et al. 2023; Guet‐McCreight et al. 2024). We then extracted exponent and offset parameters from the 1/*f* aperiodic component and maximum peak center frequency and amplitude from the periodic component. For comparison to literature values in Figure 3, we extracted corresponding average FOOOF values from Merkin et al. (2023), which compared EEG recordings across the scalp (excluding FP1, FPz, FP2, AF7, and AF8) between younger (*n* = 85; 18–35 years; 37 male) and older adults (*n* = 92; 50–86 years; 53 male). From these, we computed the expected change in FOOOF metrics relative to the mean FOOOF values of our middle‐age microcircuit models. We also performed a wavelet‐based spectrogram event analysis using the toolbox OEvents (Neymotin et al. 2022) in python, as described previously (Guet‐McCreight et al. 2024) (median threshold = 4.0, sampling rate = 40,000 Hz, window size = 24 s, minimum frequency = 1 Hz, maximum frequency = 100 Hz, frequency step = 0.5 Hz, overlapping bounding box threshold = 0.5).

### 4.8 Machine Learning Models for Classifying Changes in Cellular and Synaptic Aging Mechanisms From EEG Features

We used Tensorflow (Abadi et al. 2016) to train ANN models to classify age‐related losses in inhibitory cell type proportions, NMDA receptors, and spines. The ANN models comprised 4 input nodes, 2 × 20 hidden layer nodes with ReLU activation, and 5 output nodes with softmax activation (1 for each condition, see below). Input features included offset, exponent, peak centre frequency, and 1/*f* AUC. The model was trained to classify middle age, older, inhibitory loss, NMDA loss, and spine loss microcircuits. The Adam optimizer with Nesterov momentum and a learning rate of 0.001 was used for learning, and loss was measured using categorical cross‐entropy. Weights were initialized from a normal distribution centred at zero. All ANN models were trained using 60% of the data, validated using 10% of the data, and tested using 30% of the data. To enable a robust estimation of changes in the cellular and synaptic aging mechanisms and to assess ANN performance, we trained 50 ANN models using random permutations of the data split into 60/10/30 training/validation/testing. ANNs were trained for 100 epochs, and we enabled early stopping if validation loss did not reduce for 10 epochs. SHAP values were used to identify feature importance, where larger magnitudes indicated a larger impact on model performance (Lundberg and Lee 2017).

### 4.9 Statistics

For group comparisons we used two‐sided paired‐ and independent‐sample *t*‐tests assuming equal variance, where indicated. Cohen's *d* was calculated as follows:

$$\text{Cohe}n^{\prime }s\;d=\frac{\bar{x}-\bar{y}}{\sqrt{\frac{\left(N_{x}-1\right)\times {\sigma_{x}}^{2}+\left(N_{y}-1\right)\times {\sigma_{y}}^{2}}{N_{x}+N_{y}-2}}}$$

## Author Contributions

Alexandre Guet‐McCreight, Etienne Sibille, and Etay Hay conceived and designed the study. Alexandre Guet‐McCreight, Shreejoy Tripathy, and Etay Hay contributed to the analysis and interpretation of data. Alexandre Guet‐McCreight and Etay Hay contributed to the methodology. Alexandre Guet‐McCreight wrote and ran the simulation and analytical code. Alexandre Guet‐McCreight and Etay Hay wrote the manuscript. All authors reviewed and revised the manuscript.

## Funding

This work was supported by Canadian Institutes of Health Research, 202310AGE‐515626‐93035.

## Disclosure

Code Availability: All original code has been deposited at Zenodo and is available as of the date of publication: http://doi.org/10.5281/zenodo.15847900.

## Conflicts of Interest

The authors declare no conflicts of interest.

## Data Availability

The authors have nothing to report.

## References

[acel70329-bib-0001] Abadi, M. , A. Agarwal , P. Barham , et al. 2016. “TensorFlow: Large‐Scale Machine Learning on Heterogeneous Distributed Systems.” 10.48550/arXiv.1603.04467.

[acel70329-bib-0002] Allard, S. , T. Scardochio , A. C. Cuello , and A. Ribeiro‐da‐Silva . 2012. “Correlation of Cognitive Performance and Morphological Changes in Neocortical Pyramidal Neurons in Aging.” Neurobiology of Aging 33: 1466–1480. 21163553 10.1016/j.neurobiolaging.2010.10.011 PMC3116944

[acel70329-bib-0003] Anderson, R. M. , R. M. Glanz , S. B. Johnson , M. M. Miller , S. A. Romig‐Martin , and J. J. Radley . 2016. “Prolonged Corticosterone Exposure Induces Dendritic Spine Remodeling and Attrition in the Rat Medial Prefrontal Cortex.” Journal of Comparative Neurology 524: 3729–3746. 27113541 10.1002/cne.24027 PMC5063662

[acel70329-bib-0004] Barbaux, L. , A. A. Perrault , N. E. Cross , et al. 2025. “Effect of Chronic Benzodiazepine and Benzodiazepine Receptor Agonist Use on Sleep Architecture and Brain Oscillations in Older Adults With Chronic Insomnia.” Sleep 48: zsaf168. 40570297 10.1093/sleep/zsaf168 PMC12515599

[acel70329-bib-0005] Barzó, P. , I. Szöts , M. Tóth , et al. 2024. “Electrophysiology and Morphology of Human Cortical Supragranular Pyramidal Cells in a Wide Age Range.” eLife 13: RP100390. 10.7554/eLife.100390 PMC11952751 40152903

[acel70329-bib-0006] Bergosh, M. , S. Medvidovic , N. Zepeda , et al. 2024. “Immediate and Long‐Term Electrophysiological Biomarkers of Antidepressant‐Like Behavioral Effects After Subanesthetic Ketamine and Medial Prefrontal Cortex Deep Brain Stimulation Treatment.” Frontiers in Neuroscience 18: 1389096. 38966758 10.3389/fnins.2024.1389096 PMC11222339

[acel70329-bib-0007] Boros, B. D. , K. M. Greathouse , M. Gearing , and J. H. Herskowitz . 2019. “Dendritic Spine Remodeling Accompanies Alzheimer's Disease Pathology and Genetic Susceptibility in Cognitively Normal Aging.” Neurobiology of Aging 73: 92–103. 30339964 10.1016/j.neurobiolaging.2018.09.003 PMC6251733

[acel70329-bib-0008] Bucur, B. , and D. J. Madden . 2010. “Effects of Adult Age and Blood Pressure on Executive Function and Speed of Processing.” Experimental Aging Research 36: 153–168. 20209419 10.1080/03610731003613482 PMC2837518

[acel70329-bib-0009] Carnevale, N. T. , and M. L. Hines . 2006. The NEURON Book. Cambridge University Press.

[acel70329-bib-0010] Cesnaite, E. , P. Steinfath , M. Jamshidi Idaji , et al. 2023. “Alterations in Rhythmic and Non‐Rhythmic Resting‐State EEG Activity and Their Link to Cognition in Older Age.” NeuroImage 268: 119810. 36587708 10.1016/j.neuroimage.2022.119810

[acel70329-bib-0011] Chameh, H. M. , S. Rich , L. Wang , et al. 2021. “Diversity Amongst Human Cortical Pyramidal Neurons Revealed via Their Sag Currents and Frequency Preferences.” Nature Communications 12: 2497. 10.1038/s41467-021-22741-9 PMC8093195 33941783

[acel70329-bib-0012] Chen, Y. , E. Hunter , K. Arbabi , et al. 2023. “Robust Differences in Cortical Cell Type Proportions Across Healthy Human Aging Inferred Through Cross‐Dataset Transcriptome Analyses.” Neurobiology of Aging 125: 49–61. 36841202 10.1016/j.neurobiolaging.2023.01.013

[acel70329-bib-0013] Chinoy, E. D. , D. J. Frey , D. N. Kaslovsky , F. G. Meyer , and K. P. Wright . 2014. “Age‐Related Changes in Slow Wave Activity Rise Time and NREM Sleep EEG With and Without Zolpidem in Healthy Young and Older Adults.” Sleep Medicine 15: 1037–1045. 24980066 10.1016/j.sleep.2014.05.007 PMC4615697

[acel70329-bib-0014] Coskren, P. J. , J. I. Luebke , D. Kabaso , et al. 2015. “Functional Consequences of Age‐Related Morphologic Changes to Pyramidal Neurons of the Rhesus Monkey Prefrontal Cortex.” Journal of Computational Neuroscience 38: 263–283. 25527184 10.1007/s10827-014-0541-5 PMC4352129

[acel70329-bib-0015] Csemer, A. , A. Kovács , B. Maamrah , et al. 2023. “Astrocyte‐ and NMDA Receptor‐Dependent Slow Inward Currents Differently Contribute to Synaptic Plasticity in an Age‐Dependent Manner in Mouse and Human Neocortex.” Aging Cell 22: e13939. 37489544 10.1111/acel.13939 PMC10497838

[acel70329-bib-0016] Destexhe, A. , M. Rudolph , J.‐M. Fellous , and T. J. Sejnowski . 2001. “Fluctuating Synaptic Conductances Recreate In Vivo‐Like Activity in Neocortical Neurons.” Neuroscience 107: 13–24. 11744242 10.1016/s0306-4522(01)00344-x PMC3320220

[acel70329-bib-0017] Donoghue, T. , M. Haller , E. J. Peterson , et al. 2020. “Parameterizing Neural Power Spectra Into Periodic and Aperiodic Components.” Nature Neuroscience 23: 1655–1665. 33230329 10.1038/s41593-020-00744-x PMC8106550

[acel70329-bib-0018] Dumitriu, D. , J. Hao , Y. Hara , et al. 2010. “Selective Changes in Thin Spine Density and Morphology in Monkey Prefrontal Cortex Correlate With Aging‐Related Cognitive Impairment.” Journal of Neuroscience 30: 7507–7515. 20519525 10.1523/JNEUROSCI.6410-09.2010 PMC2892969

[acel70329-bib-0019] Engemann, D. A. , A. Mellot , R. Höchenberger , et al. 2022. “A Reusable Benchmark of Brain‐Age Prediction From M/EEG Resting‐State Signals.” NeuroImage 262: 119521. 35905809 10.1016/j.neuroimage.2022.119521

[acel70329-bib-0020] Eyal, G. , M. B. Verhoog , G. Testa‐Silva , et al. 2018. “Human Cortical Pyramidal Neurons: From Spines to Spikes via Models.” Frontiers in Cellular Neuroscience 12: 181. 30008663 10.3389/fncel.2018.00181 PMC6034553

[acel70329-bib-0021] Fuhrmann, G. , I. Segev , H. Markram , and M. Tsodyks . 2002. “Coding of Temporal Information by Activity‐Dependent Synapses.” Journal of Neurophysiology 87: 140–148. 11784736 10.1152/jn.00258.2001

[acel70329-bib-0022] Gabitto, M. I. , K. J. Travaglini , V. M. Rachleff , et al. 2024. “Integrated Multimodal Cell Atlas of Alzheimer's Disease.” Nature Neuroscience 27: 2366–2383. 39402379 10.1038/s41593-024-01774-5 PMC11614693

[acel70329-bib-0023] Gao, R. , E. J. Peterson , and B. Voytek . 2017. “Inferring Synaptic Excitation/Inhibition Balance From Field Potentials.” NeuroImage 158: 70–78. 28676297 10.1016/j.neuroimage.2017.06.078

[acel70329-bib-0024] Gavilán, M. P. , E. Revilla , C. Pintado , et al. 2007. “Molecular and Cellular Characterization of the Age‐Related Neuroinflammatory Processes Occurring in Normal Rat Hippocampus: Potential Relation With the Loss of Somatostatin GABAergic Neurons.” Journal of Neurochemistry 103: 984–996. 17666053 10.1111/j.1471-4159.2007.04787.x

[acel70329-bib-0026] Guet‐McCreight, A. , H. M. Chameh , S. Mahallati , et al. 2022. “Age‐Dependent Increased Sag Amplitude in Human Pyramidal Neurons Dampens Baseline Cortical Activity.” Cerebral Cortex 33, no. 8: 4360–4373. 10.1093/cercor/bhac348. 36124673

[acel70329-bib-0025] Guet‐McCreight, A. , H. M. Chameh , F. Mazza , et al. 2024. “In‐Silico Testing of New Pharmacology for Restoring Inhibition and Human Cortical Function in Depression.” Communications Biology 7: 1–13. 38396202 10.1038/s42003-024-05907-1 PMC10891083

[acel70329-bib-0028] Guet‐McCreight, A. , and F. K. Skinner . 2019. “Using Computational Models to Predict In Vivo Synaptic Inputs to Interneuron Specific 3 (IS3) Cells of CA1 Hippocampus That Also Allow Their Recruitment During Rhythmic States.” PLoS One 14: e0209429. 30620732 10.1371/journal.pone.0209429 PMC6324795

[acel70329-bib-0027] Guet‐McCreight, A. , and F. K. Skinner . 2020. “Computationally Going Where Experiments Cannot: A Dynamical Assessment of Dendritic Ion Channel Currents During In Vivo‐Like States.” F1000Res 9: 180. 32595950 10.12688/f1000research.22584.1 PMC7309567

[acel70329-bib-0029] Hagen, E. , S. Næss , T. V. Ness , and G. T. Einevoll . 2018. “Multimodal Modeling of Neural Network Activity: Computing LFP, ECoG, EEG, and MEG Signals With LFPy 2.0.” Frontiers in Neuroinformatics 12: 92. 30618697 10.3389/fninf.2018.00092 PMC6305460

[acel70329-bib-0030] Hawrylycz, M. J. , E. S. Lein , A. L. Guillozet‐Bongaarts , et al. 2012. “An Anatomically Comprehensive Atlas of the Adult Human Brain Transcriptome.” Nature 489: 391–399. 22996553 10.1038/nature11405 PMC4243026

[acel70329-bib-0031] Hay, E. , and I. Segev . 2015. “Dendritic Excitability and Gain Control in Recurrent Cortical Microcircuits.” Cerebral Cortex 25: 3561–3571. 25205662 10.1093/cercor/bhu200 PMC4585504

[acel70329-bib-0032] Hodge, R. D. , T. E. Bakken , J. A. Miller , et al. 2019. “Conserved Cell Types With Divergent Features in Human Versus Mouse Cortex.” Nature 573: 1–8. 10.1038/s41586-019-1506-7. PMC6919571 31435019

[acel70329-bib-0033] Kamiński, J. , S. Sullivan , J. M. Chung , I. B. Ross , A. N. Mamelak , and U. Rutishauser . 2017. “Persistently Active Neurons in Human Medial Frontal and Medial Temporal Lobe Support Working Memory.” Nature Neuroscience 20: 590–601. 28218914 10.1038/nn.4509 PMC5374017

[acel70329-bib-0034] Kauvar, I. , E. B. Richman , T. X. Liu , et al. 2025. “Conserved Brain‐Wide Emergence of Emotional Response From Sensory Experience in Humans and Mice.” Science 388: eadt3971. 40440375 10.1126/science.adt3971 PMC12286656

[acel70329-bib-0035] Khayretdinova, M. , A. Shovkun , V. Degtyarev , A. Kiryasov , P. Pshonkovskaya , and I. Zakharov . 2022. “Predicting Age From Resting‐State Scalp EEG Signals With Deep Convolutional Neural Networks on TD‐Brain Dataset.” Frontiers in Aging Neuroscience 14: 1019869. 36561135 10.3389/fnagi.2022.1019869 PMC9764861

[acel70329-bib-0036] Kim, D. , H. Jeong , J. Lee , et al. 2016. “Distinct Roles of Parvalbumin‐ and Somatostatin‐Expressing Interneurons in Working Memory.” Neuron 92: 902–915. 27746132 10.1016/j.neuron.2016.09.023

[acel70329-bib-0037] Komlósi, G. , G. Molnár , M. Rózsa , S. Oláh , P. Barzó , and G. Tamás . 2012. “Fluoxetine (Prozac) and Serotonin Act on Excitatory Synaptic Transmission to Suppress Single Layer 2/3 Pyramidal Neuron‐Triggered Cell Assemblies in the Human Prefrontal Cortex.” Journal of Neuroscience 32: 16369–16378. 23152619 10.1523/JNEUROSCI.2618-12.2012 PMC3752144

[acel70329-bib-0038] Kumral, D. , F. Şansal , E. Cesnaite , et al. 2020. “BOLD and EEG Signal Variability at Rest Differently Relate to Aging in the Human Brain.” NeuroImage 207: 116373. 31759114 10.1016/j.neuroimage.2019.116373

[acel70329-bib-0039] Lee, S. , I. Kruglikov , Z. J. Huang , G. Fishell , and B. Rudy . 2013. “A Disinhibitory Circuit Mediates Motor Integration in the Somatosensory Cortex.” Nature Neuroscience 16: 1662–1670. 24097044 10.1038/nn.3544 PMC4100076

[acel70329-bib-0040] Legge, G. E. , C. Granquist , A. Lubet , R. Gage , and Y.‐Z. Xiong . 2019. “Preserved Tactile Acuity in Older Pianists.” Attention, Perception, & Psychophysics 81: 2619–2625. 10.3758/s13414-019-01844-y PMC6858535 31410761

[acel70329-bib-0041] Legge, G. E. , C. Madison , B. N. Vaughn , A. M. Y. Cheong , and J. C. Miller . 2008. “Retention of High Tactile Acuity Throughout the Life Span in Blindness.” Perception & Psychophysics 70: 1471–1488. 19064491 10.3758/PP.70.8.1471 PMC3611958

[acel70329-bib-0042] Lundberg, S. M. , and S.‐I. Lee . 2017. “A Unified Approach to Interpreting Model Predictions in Advances in Neural Information Processing Systems vol. 30 Curran Associates, Inc.”

[acel70329-bib-0043] Mäki‐Marttunen, T. , F. Krull , F. Bettella , et al. 2019. “Alterations in Schizophrenia‐Associated Genes Can Lead to Increased Power in Delta Oscillations.” Cerebral Cortex 29: 875–891. 30475994 10.1093/cercor/bhy291 PMC6319172

[acel70329-bib-0044] Mazza, F. , A. Guet‐McCreight , T. A. Valiante , J. D. Griffiths , and E. Hay . 2023. “In‐Silico EEG Biomarkers of Reduced Inhibition in Human Cortical Microcircuits in Depression.” PLoS Computational Biology 19: e1010986. 37036854 10.1371/journal.pcbi.1010986 PMC10085061

[acel70329-bib-0045] McCann, H. , G. Pisano , and L. Beltrachini . 2019. “Variation in Reported Human Head Tissue Electrical Conductivity Values.” Brain Topography 32: 825–858. 31054104 10.1007/s10548-019-00710-2 PMC6708046

[acel70329-bib-0046] McKinney, B. C. , C. W. Lin , H. Oh , G. C. Tseng , D. A. Lewis , and E. Sibille . 2015. “Hypermethylation of BDNF and SST Genes in the Orbital Frontal Cortex of Older Individuals: A Putative Mechanism for Declining Gene Expression With Age.” Neuropsychopharmacol 40: 2604–2613. 10.1038/npp.2015.107 PMC4569950 25881116

[acel70329-bib-0047] Merkin, A. , S. Sghirripa , L. Graetz , et al. 2023. “Do Age‐Related Differences in Aperiodic Neural Activity Explain Differences in Resting EEG Alpha?” Neurobiology of Aging 121: 78–87. 36379095 10.1016/j.neurobiolaging.2022.09.003

[acel70329-bib-0048] Montemurro, S. , D. Borek , D. Marinazzo , et al. 2024. “Aperiodic Component of EEG Power Spectrum and Cognitive Performance Are Modulated by Education in Aging.” Scientific Reports 14: 15111. 38956186 10.1038/s41598-024-66049-2 PMC11220063

[acel70329-bib-0049] Mullinger, K. J. , J. Havenhand , and R. Bowtell . 2013. “Identifying the Sources of the Pulse Artefact in EEG Recordings Made Inside an MR Scanner.” NeuroImage 71: 75–83. 23313417 10.1016/j.neuroimage.2012.12.070 PMC3601330

[acel70329-bib-0050] Neymotin, S. A. , I. Tal , A. Barczak , et al. 2022. “Detecting Spontaneous Neural Oscillation Events in Primate Auditory Cortex.” eNeuro 9: ENEURO.0281‐21.2022. 10.1523/ENEURO.0281-21.2022 PMC9395248 35906065

[acel70329-bib-0051] Nuwer, J. L. , N. Povysheva , and T. C. Jacob . 2023. “Long‐Term α5 GABA A Receptor Negative Allosteric Modulator Treatment Reduces NMDAR‐Mediated Neuronal Excitation and Maintains Basal Neuronal Inhibition.” Neuropharmacology 237: 109587. 37270156 10.1016/j.neuropharm.2023.109587 PMC10527172

[acel70329-bib-0052] Obermayer, J. , T. S. Heistek , A. Kerkhofs , et al. 2018. “Lateral Inhibition by Martinotti Interneurons Is Facilitated by Cholinergic Inputs in Human and Mouse Neocortex.” Nature Communications 9: 1–14. 10.1038/s41467-018-06628-w PMC6173769 30291244

[acel70329-bib-0053] Park, J. , R. L. M. Ho , W. Wang , V. Q. Nguyen , and S. A. Coombes . 2024. “The Effect of Age on Alpha Rhythms in the Human Brain Derived From Source Localized Resting‐State Electroencephalography.” NeuroImage 292: 120614. 38631618 10.1016/j.neuroimage.2024.120614

[acel70329-bib-0054] Park, J. , R. L. M. Ho , W. E. Wang , S. Y. Chiu , Y. S. Shin , and S. A. Coombes . 2025. “Age‐Related Changes in Neural Oscillations Vary as a Function of Brain Region and Frequency Band.” Frontiers in Aging Neuroscience 17: 1488811. 40040743 10.3389/fnagi.2025.1488811 PMC11876397

[acel70329-bib-0055] Pegasiou, C. M. , A. Zolnourian , D. Gomez‐Nicola , et al. 2020. “Age‐Dependent Changes in Synaptic NMDA Receptor Composition in Adult Human Cortical Neurons.” Cerebral Cortex 30: 4246–4256. 32191258 10.1093/cercor/bhaa052

[acel70329-bib-0056] Peich, M.‐C. , M. Husain , and P. M. Bays . 2013. “Age‐Related Decline of Precision and Binding in Visual Working Memory.” Psychology and Aging 28: 729–743. 23978008 10.1037/a0033236 PMC3913749

[acel70329-bib-0057] Petanjek, Z. , M. Judaš , G. Šimić , et al. 2011. “Extraordinary Neoteny of Synaptic Spines in the Human Prefrontal Cortex.” Proceedings of the National Academy of Sciences 108: 13281–13286. 10.1073/pnas.1105108108 PMC3156171 21788513

[acel70329-bib-0058] Pi, H.‐J. , B. Hangya , D. Kvitsiani , J. I. Sanders , Z. J. Huang , and A. Kepecs . 2013. “Cortical Interneurons That Specialize in Disinhibitory Control.” Nature 503: 521–524. 24097352 10.1038/nature12676 PMC4017628

[acel70329-bib-0059] Ponce, M. , R. van Zon , S. Northrup , et al. 2019. “Deploying a Top‐100 Supercomputer for Large Parallel Workloads: The Niagara Supercomputer.” In Proceedings of the Practice and Experience in Advanced Research Computing on Rise of the Machines (Learning), 1–8. Association for Computing Machinery. 10.1145/3332186.3332195.

[acel70329-bib-0060] Prevot, T. D. , A. Sumitomo , T. Tomoda , et al. 2021. “Reversal of Age‐Related Neuronal Atrophy by α5‐GABAA Receptor Positive Allosteric Modulation.” Cerebral Cortex 31: 1395–1408. 33068001 10.1093/cercor/bhaa310 PMC7786363

[acel70329-bib-0061] Reimann, M. W. , C. A. Anastassiou , R. Perin , S. L. Hill , H. Markram , and C. Koch . 2013. “A Biophysically Detailed Model of Neocortical Local Field Potentials Predicts the Critical Role of Active Membrane Currents.” Neuron 79: 375–390. 23889937 10.1016/j.neuron.2013.05.023 PMC3732581

[acel70329-bib-0062] Rezaei, S. , T. D. Prévot , E. Vieira , and E. Sibille . 2024. “LPS‐Induced Inflammation Reduces GABAergic Interneuron Markers and Brain‐Derived Neurotrophic Factor in Mouse Prefrontal Cortex and Hippocampus.” Brain, Behavior, & Immunity‐Health 38: 100761. 10.1016/j.bbih.2024.100761 PMC10992730 38586282

[acel70329-bib-0063] Roopun, A. K. , S. J. Middleton , M. O. Cunningham , et al. 2006. “A beta2‐Frequency (20–30 Hz) Oscillation in Nonsynaptic Networks of Somatosensory Cortex.” PNAS 103: 15646–15650. 17030821 10.1073/pnas.0607443103 PMC1592532

[acel70329-bib-0064] Rosanally, S. , F. Mazza , H. K. Yao , and E. Hay . 2024. “Linking Reduced Prefrontal Microcircuit Inhibition in Schizophrenia to EEG Biomarkers In Silico.” bioRxiv: 2023.08.11.553052. 10.1101/2023.08.11.553052. PMC13229350 42228689

[acel70329-bib-0065] Salvatore, S. V. , P. M. Lambert , A. Benz , et al. 2024. “Periodic and Aperiodic Changes to Cortical EEG in Response to Pharmacological Manipulation.” Journal of Neurophysiology 131: 529–540. 38323322 10.1152/jn.00445.2023 PMC11305649

[acel70329-bib-0066] Schmidt, F. , S. K. Danböck , E. Trinka , D. P. Klein , G. Demarchi , and N. Weisz . 2025. “Age‐Related Changes in “Cortical” 1/f Dynamics Are Linked to Cardiac Activity.” eLife 13: RP100605. 41037316 10.7554/eLife.100605 PMC12490856

[acel70329-bib-0067] Seeman, S. C. , L. Campagnola , P. A. Davoudian , et al. 2018. “Sparse Recurrent Excitatory Connectivity in the Microcircuit of the Adult Mouse and Human Cortex.” eLife 7: e37349. 30256194 10.7554/eLife.37349 PMC6158007

[acel70329-bib-0068] Smith, A. E. , A. Chau , D. Greaves , H. A. D. Keage , and D. Feuerriegel . 2023. “Resting EEG Power Spectra Across Middle to Late Life: Associations With Age, Cognition, APOE‐ɛ4 Carriage, and Cardiometabolic Burden.” Neurobiology of Aging 130: 93–102. 37494844 10.1016/j.neurobiolaging.2023.06.004

[acel70329-bib-0069] Tomoda, T. , A. Sumitomo , D. Newton , and E. Sibille . 2022. “Molecular Origin of Somatostatin‐Positive Neuron Vulnerability.” Molecular Psychiatry 27: 2304–2314. 35145229 10.1038/s41380-022-01463-4 PMC9133093

[acel70329-bib-0070] Vardalaki, D. , K. Chung , and M. T. Harnett . 2022. “Filopodia Are a Structural Substrate for Silent Synapses in Adult Neocortex.” Nature 612: 323–327. 36450984 10.1038/s41586-022-05483-6

[acel70329-bib-0071] Wang, M. , N. J. Gamo , Y. Yang , et al. 2011. “Neuronal Basis of Age‐Related Working Memory Decline.” Nature 476: 210–213. 21796118 10.1038/nature10243 PMC3193794

[acel70329-bib-0072] Welch, P. 1967. “The Use of Fast Fourier Transform for the Estimation of Power Spectra: A Method Based on Time Averaging Over Short, Modified Periodograms.” IEEE Transactions on Audio and Electroacoustics 15: 70–73.

[acel70329-bib-0073] Yao, H. K. , A. Guet‐McCreight , F. Mazza , et al. 2022. “Reduced Inhibition in Depression Impairs Stimulus Processing in Human Cortical Microcircuits.” Cell Reports 38: 110232. 35021088 10.1016/j.celrep.2021.110232

[acel70329-bib-0074] Yao, H. K. , F. Mazza , T. D. Prevot , E. Sibille , and E. Hay . 2025. “Spine Loss in Depression Impairs Dendritic Signal Integration in Human Cortical Microcircuit Models.” iScience 28: 112136. 40292322 10.1016/j.isci.2025.112136 PMC12032932

[acel70329-bib-0075] Zhang, J. , J. Xia , H. Zhou , and S. Wang . 2025. “Gamma Synchronization Between the Medial Temporal Lobe and Medial Frontal Cortex for Goal‐Directed Visual Attention in Humans.” Cell Reports 44: 115905. 40570370 10.1016/j.celrep.2025.115905 PMC12781120

